# Complete Genome Sequence of Bacillus subtilis Strain DKU_NT_03, Isolated from a Traditional Korean Food Using Soybean (Chung-gook-jang) for High-Quality Nattokinase Activity

**DOI:** 10.1128/genomeA.00526-18

**Published:** 2018-06-21

**Authors:** Hee-Won Jeong, Man-Seok Bang, Yea-jin Lee, Su Ji Lee, Sang-Cheol Lee, Jang-In Shin, Chung-Hun Oh

**Affiliations:** a Department of Medical Laser, Graduate School, Dankook University, Cheonan, Choongnam, Republic of Korea; b Department of Oral Physiology, College of Dentistry, Dankook University, Cheonan, Choongnam, Republic of Korea

## Abstract

We present here the complete genome sequence of Bacillus subtilis strain DKU_NT_03 isolated from the traditional Korean food chung-gook-jang, which is made from soybeans. This strain was chosen to identify genetic factors with high-quality nattokinase activity.

## GENOME ANNOUNCEMENT

Chung-gook-jang is a traditional fermented Korean food that is made from soybeans and thus contains many proteins. The fermentation of the soybeans also produces a viscous, physiologically active substance (1, 2). This substance has been reported to be useful for its antimicrobial, thrombolytic, and immunological activities and, as a result, chung-gook-jang has been attracting attention as a functional food (3–7). This study was conducted to obtain strains with proteolytic capacity and viscosity, which are two of the most important criteria for producing high-quality fermented foods. We found a Bacillus subtilis strain, DKU_NT_03, that produces viscous material with high efficiency.

B. subtilis strain DKU_NT_03 was cultivated from traditional Korean food using soybean (chung-gook-jang) and LB agar. Samples were grown and maintained at 37°C in LB broth overnight until they were axenic. DNA was then extracted using the Wizard genomic DNA purification kit (Promega) following the manufacturer’s instructions. Whole-genome sequencing was performed on the PacBio RS II sequencing platform (Pacific Biosciences, USA) at Macrogen (Seoul, Republic of Korea) (8).

Sequencing libraries from the genomic DNA extracts were prepared using SMRT Cell 8Pac version 3.0 and the DNA polymerase binding kit P6 and were sequenced using PacBio RS II technology (Pacific Biosciences).

The 113,728 PacBio reads of B. subtilis strain DKU_NT_03 were assembled using the Hierarchical Genome Assembly Process version 3.0 (HGAP3) protocol, and the ends of each contig were overlapped to one circular chromosome, which comprised 4,196,031 bp with a GC content of 43.3% and an average sequencing depth of 203× (9–11). The genomes were annotated with Prokka software, and functional categories were predicted with the Rapid Annotations using Subsystems Technology (RAST) version 2.0 server (12, 13). The chromosome contains 4,369 coding sequences, 87 tRNAs, and 30 rRNAs. A plasmid was not found in this strain.

### Accession number(s).

The complete genome sequence of B. subtilis strain DKU_NT_03 was deposited in GenBank under the accession number CP022891.
